# What shapes local health system actors’ thinking and action on social inequalities in health? A meta-ethnography

**DOI:** 10.1057/s41285-022-00176-6

**Published:** 2022-01-31

**Authors:** Naoimh E. McMahon

**Affiliations:** Division of Health Research, National Institute for Health Research School for Public Health Research (NIHR SPHR), Lancaster University, Lancaster, LA1 4YW UK

**Keywords:** Social inequalities in health, Social determinants of health, Discourse, Meta-ethnography, Health systems

## Abstract

Local health systems are increasingly tasked to play a more central role in driving action to reduce social inequalities in health. Past experience, however, has demonstrated the challenge of reorienting health system actions towards prevention and the wider determinants of health. In this review, I use meta-ethnographic methods to synthesise findings from eleven qualitative research studies that have examined how ambitions to tackle social inequalities in health take shape within local health systems. The resulting line-of-argument illustrates how such inequalities continue to be problematised in narrow and reductionist ways to fit both with pre-existing conceptions of health, and the institutional practices which shape thinking and action. Instances of health system actors adopting a more social view of inequalities, and taking a more active role in influencing the social and structural determinants of health, were attributed to the beliefs and values of system leaders, and their ability to push-back against dominant discourses and institutional norms. This synthesised account provides an additional layer of understanding about the specific challenges experienced by health workforces when tasked to address this complex and enduring problem, and provides essential insights for understanding the success and shortcomings of future cross-sectoral efforts to tackle social inequalities in health.

**Supplementary Information:**

The online version contains supplementary material available at 10.1057/s41285-022-00176-6.

## Introduction

Social inequalities in health are differences in health outcomes that exist between groups of different socioeconomic position. In contrast to differences that arise due to factors such as ageing or chance, these inequalities are said to be “*systematic, socially produced* (and therefore modifiable) and *unfair”* (Whitehead and Dahlgren 2006). They reflect the conditions in which people are born, grow, live, work, and age; conditions that are in turn shaped by the grossly inequitable distribution of power and resources in society (Marmot et al. 2008). These underlying or ‘root’ causes of social inequalities in health have, typically, been considered to be beyond the purview of local health systems. However, greater recognition of the financial cost of socioeconomic inequality (Asaria et al. 2016), along with the rising demand on services due to preventable ill-health (National Health Service 2019), has meant that health systems are increasingly mandated to play a more central role in addressing them. For example, in England, it is envisioned that ongoing reforms will enable local health systems to reach beyond traditional health services and work alongside local authorities and voluntary organisations to drive action on the social and economic determinants of health (The King's Fund 2021). Previous research, however, has demonstrated how difficult it is to reorient health system efforts towards more preventative action, and towards tackling the social and structural drivers of inequalities in health (e.g. Blackman et al. 2012; Orton et al. 2011). Current ambitions are also set against an especially challenging backdrop where, after over a decade of austerity and cuts to public services, life expectancy improvements in England have stalled (Marmot et al. 2020a), and widening inequalities are now being further exacerbated by the inequitable impacts of the ongoing Covid-19 pandemic (Marmot et al. 2020b).

Further qualitative research has been conducted in recent years, both in the UK and internationally, to explore how this mandate to tackle health inequalities and their wider determinants takes shape within local health systems. These studies have a particular focus on illuminating how and why individual actors think about, and work to address, health inequalities in the ways that they do (e.g. general practitioners, public health officers, health system leaders etc.). In this review, I draw on meta-ethnographic methods to synthesise findings from these investigations into a novel, overarching, and theoretically informed ‘line-of-argument’ about what sustains the gap between the recognised need for greater preventative action on the underlying causes of inequalities, and what ultimately gets implemented in practice. This synthesised account provides an additional layer of insight about the specific challenges experienced by health system workforces when tasked to address this extremely complex and enduring problem. These insights which will be essential to understanding the future success and shortcomings of reforms, both in the UK and internationally, that are designed to enable cross-sectoral and collaborative action to reduce social inequalities in health.

## Methods

The review is reported in line with the eMERGe guidance for reporting meta-ethnography (France et al. 2019a), which aligns to Noblit and Hare’s 7 stages (Noblit and Hare 1988): (i) getting started, (ii) deciding what is relevant, (iii) reading the studies, (iv) determining how the studies are related, (v) translating the studies into each other, (vi) synthesising translations, and (vii) expressing the synthesis (see Appendix A). As the details of the aims and rationale for the review have already been described (Stage 1), this section begins with Stage 2: Deciding what is relevant.

### Deciding what is relevant

Stage 2 involved extensive reading of potentially relevant studies to familiarise myself with the volume of available literature, and the different ways in which authors have tried to unpack the puzzle of why health system actions often diverge from stated intentions to reduce inequalities through action on their underlying causes. Studies were deemed to be relevant to the review if they: (i) provided in-depth explanatory accounts about how health system actors come to both think about, and act upon, social inequalities in health, (ii) were published in English, and (iii) in a peer-reviewed journal. Studies which focused solely on inequalities in access to healthcare were excluded.

**Fig. 1:**
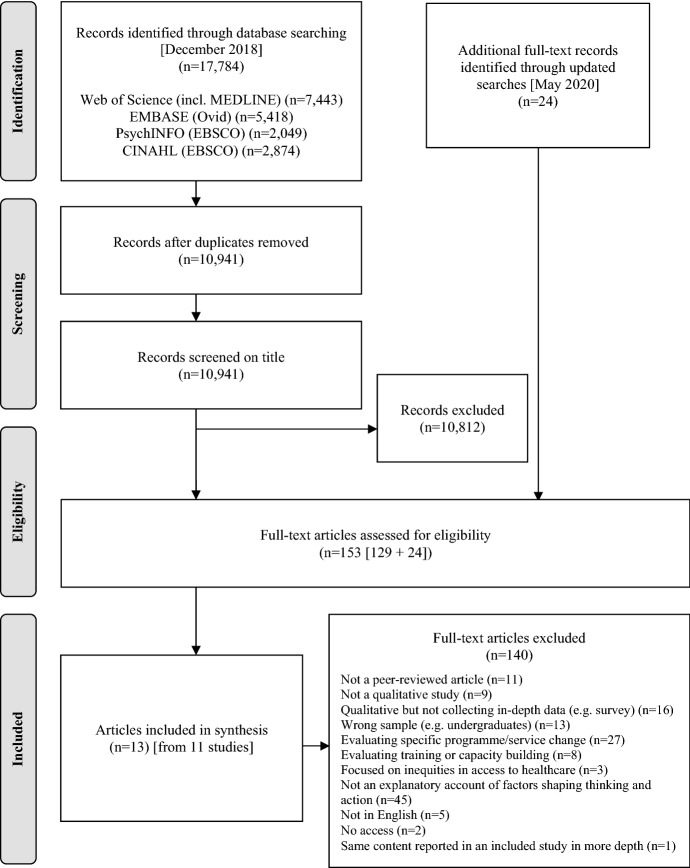
PRISMA flow diagram

Searches were performed at two time points. Four electronic databases were initially searched from their inception to the 11th of December 2018 (Web of Science including MEDLINE; PsycINFO; EMBASE and CINAHL). The full search strategy was rerun on the 22nd of May 2020. The search string was devised using target papers, and included a combination of terms for inequalities in health and qualitative research: ("health inequalit*" or "health equit*" or "social determinants of health”) AND (qualitative or interview* or focus group* or discourse* or framing* or construct* or perception* or perspective* or understand* or discussion*). Hand-searching of reference lists and citation tracking was also carried out for all included articles. Retrieved citations were compiled into a single EndNote® library, screened on title and abstract, and for those deemed to be potentially relevant, full texts were retrieved and assessed for eligibility. The flow of papers through this process is shown in Fig. 1, and an overview of the included studies is provided in Table 1 (organised chronologically by year of data collection). A list of the excluded citations with reasons is provided in Appendix B in the online supplement.

**Table 1:** Characteristics of included studies

ID	Lead author (year)	Country	Aims	Theoretical perspective/approach	Sample and data collection
1	Blackman et al. (2009)	Great Britain	To examine and compare the health inequalities discourses of local actors in England, Scotland, and Wales in the context of national differences in local governance, performance assessment, and targets	Not explicitly stated	Interviews with 130 senior figures at a local strategic level in the NHS, local government and various local partnerships [Jun–Aug 2006]
	Blackman et al. (2012)	Great Britain	To compare how the national circumstances of a problem (i.e. health inequalities) affects how it is framed, and how this is reflected in the narratives of those responsible for local implementation	Frame-reflexive discourse	Semi-structured face-to-face interviews with 197 senior figures at a local strategic level in the National Health Service (NHS), local government and various local partnerships [2006 and 2008]
2	Orton et al. (2011)	England	To examine the experiences of those involved in decision-making to reduce health inequalities, using cardiovascular disease as a case study	Grounded theory	Qualitative interviews and focus group discussions with 40 decision-makers in various public health roles [no date]
3	McIntyre et al. (2013)	Canada	To discern the reasons for limited action by examining perceptions of the social determinants of health	Discourse analysis	Discussions with two groups: (i) 50 community health workers and (ii) 12 child and youth advocacy organisation members [Aug – Nov 2009]
4	Mead et al. (2020)	England	To explore public health policy implementation by examining how local actors make sense of and work to address social inequalities in health	Figurational sociology (Elias 1978)	Ethnographic case study including interviews and focus groups with 31 professionals working in a public sector partnership [Apr 2010–Sept 2011]
5	Brassolotto et al. (2014)	Canada	To examine the worldviews of public health officials to understand how differences in action on the social determinants of health come about	Bachelard’s epistemological obstacles (Tiles 1984) and social determinants of health discourses (Raphael 2011)	Interviews with 18 public health officials from 9 Ontario Public Health Units (PHU) demonstrating various degrees of social determinants of health activity [Spring–Summer 2011]
	Raphael and Brassolotto (2015)	Canada	To illuminate the factors that shape local public health unit action on the social determinants of health	Critical realism	Interviews with 18 public health officials from 9 Ontario PHUs demonstrating various degrees of social determinants of health activity [Spring–Summer 2011]
6	Warwick‐Giles et al. (2017)	England	To explore how newly formed clinical commissioning groups made sense of their new ‘duty’ to tackle health inequalities	Sensemaking (Weick 1995)	Interviews with 21 governing members of 3 CCGs [Jan 2012–Dec 2012]
7	Pauly et al. (2017)	Canada	To study the application of a health equity lens by senior leaders during a time of health system renewal	Intersectionality, complexity science, and social justice theories	Semi-structured qualitative focus groups and interviews with 55 senior leaders from six health authorities and the provincial Ministry of Health [2013/2014]
8	Exworthy and Morcillo (2019)	England	To examine general practitioners’ knowledge of, and their role, in tackling health inequalities, in relation to their professional responsibilities	Model of physician responsibility (Gruen et al. 2004)	Interviews with 13 GPs [2013/2014]
9	Babbel et al. (2017)	Scotland	To explore how general practitioners understand the fundamental causes of health inequalities, and how they conceptualise their role in mitigating these	Social determinants of health discourses (Raphael 2011) and structural competency (Metzl and Hansen 2014)	Semi-structured interviews 24 General Practitioners (some of whom worked in the most deprived areas in Scotland) [Dates of interviews not provided]
10	Mackenzie et al. (2017)	Scotland	To understand the implicit theories of health inequalities of both practitioners and policymakers working within a single health care system	Social determinants of health discourses (Raphael 2011)	10 semi‐structured interviews with participants representing the Scottish Government, Scotland's special health board for health improvement, and from planning and practitioner roles within primary care and health policy [no date]
11	Javanparast et al. (2018)	Australia	To explore how institutional factors, ideas, and actors condition and constrain population health planning within Australian primary health care	Institutional theory	Individual telephone interviews with 50 senior staff of primary care institutions (Medicare locals) [2014/2015]

Although a significant number of qualitative studies were identified during the searches, a large majority of these either examined the implementation of specific initiatives to reduce social inequalities in health (e.g. area-based initiatives; Health in All Policies) or were found not to provide explanatory insights about health system actors’ perspectives and practices*.* While there were no limits on country or setting, the inclusion criteria, and perhaps also how the review question was framed, resulted in a set of studies from English speaking nations which were most recognisably situated within a social inequalities in health or social determinants of health research tradition. Three of the included studies centred on a single professional group (e.g. general practitioners), with the remainder including participants from a mix of professions or spanning front-line, operational, and strategic leadership roles.

### Reading and data extraction

Stage 3 involved the repeated reading of included studies to become familiar with their content, and to make a note of author interpretations relevant to the aims of the review. In meta-ethnography, a distinction is often made between three levels of interpretation. Participants’ own views and perspectives are treated as ‘1st order’ constructs. Study authors’ interpretations of these 1st order constructs are considered ‘2nd order’ constructs. Lastly, ‘3rd order’ constructs refer to the new insights and interpretations generated by review authors through the process of synthesising the included studies (Malpass et al. 2009). Akin to the experience of others (for example Atkins et al. 2008; Smith and Anderson 2017), I found it difficult to differentiate between 1st and 2nd order constructs in the published articles, as participant quotes were predominantly used to evidence authors’ own interpretations. As such, all data extracted at this stage were treated as 2nd order constructs. Further contextual details were also collected using a bespoke data extraction form (e.g. author details, year of publication, country, study aims, sample, method of data collection, and the approach to analysis). In light of the difficulty of differentiating a poor-quality study from a poorly reported study (Atkins et al. 2008), a formal approach to quality appraisal was not used. However, only studies which had undergone peer-review were eligible for inclusion.

### Analysis and synthesis

Stage 4 of a meta-ethnography involves looking across the 2nd order constructs for each study to establish how the studies are related to each other. This process was supported by developing a table in Microsoft Word® with the included articles forming the first row, and the 2nd order constructs forming the columns. In a process described as ‘translation’ (Noblit and Hare 1988), I worked systematically through each 2nd order construct to examine how it might be related to those identified in the other studies. It became evident during this process that the articles could be grouped into two clusters, those which focused more on the influence of organisational factors, and those which emphasised the intrinsic characteristics of individual health system actors. In line with the guidance from France et al. (2019b), I first carried out a reciprocal translation for the 2nd order constructs of each cluster (Stage 5), before bringing these together and synthesising into a ‘line-of-argument’, or a ‘new storyline’, about what the studies say (Stage 6). As Thorne et al. (2004) describes, this process is based on the premise ‘that often people study different aspects of phenomena’, and that by arranging the translated 2nd order constructs in a particular way, it is ‘allows us to construct an argument about what the set of ethnographies say’.

After some trial and error, I found that arranging the study insights into a storyline underpinned by Foucauldian ideas provided the most useful and meaningful account of the data. Taking guidance from the structured approaches to Foucauldian-inspired analysis offered by Bacchi (2009) and Willig (2013), the line-of-argument starts by detailing how social inequalities in health tended to be ‘problematised’ or ‘discursively constructed’ by participants within the included studies. I then locate these perspectives within the wider discourses from which they arise, and go on to illuminate how such discourses have very real and material effects in terms of constraining how health system actors can both think about, and work to address, social inequalities in health. A Foucauldian perspective proved especially useful in this review because it makes explicit how health system actors are governed by the *problems* they are tasked to address, and how these problems are shaped by powerful and influential discourses operating both within and outside of the health system.

## Findings

Eleven studies, reported in thirteen published articles, were included in the meta-ethnography (Table 1). The earliest studies were conducted in Great Britain where authors sought to illuminate how the national policy imperative to reduce health inequalities took shape within local health systems between 2006 and 2010 (Blackman et al. 2009; Blackman et al. 2012; Orton et al. 2011). These were followed by a number of qualitative studies in the UK and Canada which specifically set out to illustrate how individual health system actors problematised health inequalities and action on the social determinants of health (McIntyre et al. 2013; Mead et al. 2020; Brassolotto et al. 2014; Raphael and Brassolotto 2015; Pauly et al. 2017; Exworthy and Morcillo 2019; Babbel et al. 2017; Mackenzie et al. 2017). Two additional studies, one from England (Warwick‐Giles et al. 2017) and one from Australia (Javanparast et al. 2018) were also included, with each contributing further insights on how health equity objectives were operationalised during more recent health system reforms. There was substantial variation in the level of detail provided in the included studies about how individual participants problematised social inequalities in health. Some provided a quantified breakdown of perspectives, either at the level of the individual practitioner (Babbel et al. 2017), or organisation (Brassolotto et al. 2014), whereas others relied on more high-level generalisations (e.g. Blackman et al. 2009; Blackman et al. 2012; McIntyre et al. 2013). Taken together however, the studies collectively point to the existence of a predominant perspective on social inequalities in health.

### A predominant perspective on social inequalities in health

This predominant perspective was characterised by a concern for the health of specific ‘disadvantaged’ groups or geographical regions. While instances of participants drawing on ‘victim blaming’ discourses, or having an expressly negative view of such groups (and their lifestyle ‘choices’) were rare, health system actors were generally found to hold an ‘individualised’ understanding of inequalities in health. For example, although they often acknowledged the importance of the social determinants in shaping health outcomes, these determinants were invariably problematised as ‘individual risk factors’ for health, consequently ‘obscuring’ their relationship to structural inequality, politics, and policy (Mead et al. 2020; Brassolotto et al. 2014). The result was a tendency for health system actors to focus on activities to increase *individual* access to, or uptake of, health promoting resources (i.e. healthcare, healthy lifestyles, and the wider determinants of health), rather than questioning or challenging their inequitable distribution. As such, the majority were said to hold a ‘reductionist’ view of the problem, which failed to account for both the ‘complexity’ of health inequalities and their ‘political roots’ (Pauly et al. 2017; McIntyre et al. 2013; Mackenzie et al. 2017). In stark contrast were the minority of study participants who problematised inequalities in terms of a social gradient in health, and who viewed the distribution of the social determinants as indicative of wider structural inequality. Thus, rather than a cleaving apart of forces, these individuals were found to explicitly ‘tie together’ individual health outcomes, the social determinants of health, politics, and policy. While all studies alluded to the importance of individual perspectives, Babbel et al. (2017) and Brassolotto et al. (2014) were most explicit in arguing for the existence of ‘clear linkages’ between actors’ problematisations of health inequalities and perceptions about their own role in taking action. Those who subscribed to narrower versions of the problem were inclined to see tackling wider social forces as ‘outside the scope’ of their work, in contrast to the minority who felt they were uniquely well-placed, and indeed had a professional responsibility, to try and influence determinants beyond individual encounters.

### The role of discourse in sustaining the predominant perspective

This predominant perspective on social inequalities in health was found to be shaped, and sustained, by powerful and influential discourses operating both within and outside of local health systems. Drawing on the translated 2nd order constructs (Table 2) and key examples from the included articles, the following sections detail the role of discourse in shaping individual conceptions of health, how these tend to align with internal governance arrangements and external pressures on local health systems, and consequently why it proves so difficult for health equity ‘counter-discourses’ to gain traction in practice.

**Table 2:** Translated 2nd order constructs that explain the predominant perspective on social inequalities in health

Organising categories	2nd order constructs	Relevant studies
Individual sense-making	Dominant discourses result in epistemological barriers to adopting a more social view of health	3, 4, 5, 11
	Local health data promotes dichotomous framing of inequality and a focus on the ‘worst off’ places and populations	4, 7
	Assigning a label to specific groups (e.g. vulnerable) implicitly locates the problem within individuals or places	4, 7
	Narrow perspectives reinforced by national policy framings of health inequalities (e.g. targeted action to reduce the gap)	1, 2
	Impact of targeted interventions based on assumptions of benefit rather than evidence, or clear causal links	1, 7, 8
	Simplifying the problem of inequalities in health (e.g. a focus on access) reflects the need to identify practical actions	3, 4, 5, 6, 7
	Individual perspectives reflect past personal exposure to inequality and exposure to broader social concepts	5, 9
	Individual perspectives reflect personal political perspectives, and related social empathy towards patients/populations	9
	Senior leaders can ‘push against’ dominant discourses and successfully shift individual/organisational thinking and action	5, 11
Organisational influences	Performance indicators create pressure in the system to address those targets which ‘shout the loudest’	1, 2, 6, 8, 11
	Addressing health inequalities considered to be a ‘Cinderella area’, easily eclipsed by other priorities	1, 2, 11
	Unrealistic timeframes to make an impact against targets leads to the ‘medicalising’ of health inequalities	1, 2, 11
	Alignment between regulatory frameworks and biomedical perspectives constrains long-term planning	11
	Actions to reduce health inequalities reframed in more neutral terms to fit with institutional norms and objectives	5, 7, 11
	Complex causal chains for actions targeting health inequalities are a poor fit with cultures of evidence-based practice	1, 2
	Reorganisations continuously disrupt the working-relationships needed for long-term partnerships and action	1, 6, 11
	Significant variation in organisational capability (e.g. knowledge) limit longer-term action on wider determinants	6, 11
	Hospitals and medical professionals powerfully influence health system planning and decision-making	1, 11
	Organisations shared histories of working together can positively and negatively shape collective understanding and action	6
External pressures	‘Popular’ understandings of health shaped and reinforced by media and political ‘preoccupation’ with acute care	1, 2,
	Pressure for health system actors to be apolitical constrains advocacy on inequalities in health	5, 7
	Lack of ‘bottom-up’ pressure to prioritise action to reduce social inequalities in health	1

### Biomedical individualism and positivist conceptions of health

Authors attributed the predominant perspective on social inequalities in health to the influence of biomedical, individualistic, and positivist discourses that are so prominent in Western societies (McIntyre et al. 2013; Mead et al. 2020; Brassolotto et al. 2014; Javanparast et al. 2018). Collectively, these discourses operate to promote a focus on individuals (rather than the contexts and conditions in which they live); to emphasise the importance of individual responsibility for health; and to privilege causal understandings that hinge on direct and observable logics of cause and effect. Brassolotto et al. (2014) were most explicit in deeming the different perspectives of public health unit staff in their study as being ‘epistemological’ in nature, arguing that it was the different worldviews of participants which served to either exclude, or bring into the frame of understanding, the less visible social and structural forces that drive and sustain inequalities in health. Two studies went a step further in trying to account for why some health system actors, albeit a minority, rejected the dominant discourses to problematise health inequalities through a more ‘structural’, rather than individualised, lens. The concept of ‘exposure’ was central to these accounts. Raphael and Brassolotto (2015) highlight how some public health unit staff held a greater awareness and sensitivity towards structural factors because of either ‘first-hand’ experience of inequalities (e.g. through socioeconomic background, ethnicity), or because they had initially trained in non-medical fields (e.g. social work, political science). In a similar way, Babbel et al. (2017) found that the general practitioners in their study who held more structural perspectives had more experience of working in areas of severe deprivation, and as a result demonstrated great ‘social empathy’ towards patients and the wider forces impacting upon their personal circumstances. The authors do note however that the direction of this relationship is unclear and that it may be the case that GPs with a ‘particular political perspective’ gravitate towards such roles.

### Alignment with organisational discourses and external pressures

Importantly, the predominant perspective on social inequalities in health was also shown to both closely align with, and be further reinforced by, organisational discourses. Firstly, study authors illustrated how the very practice of using epidemiological data to categorise and compare inequalities between population groups and geographical regions (e.g. life expectancy figures), served to unhelpfully promote dichotomous extremes and a focus amongst participants on the ‘worst off’ groups (Mead et al. 2020). As Pauly et al. (2017) further describe, the prominent practice of assigning a ‘label’ to populations (e.g. ‘at risk’, ‘vulnerable’) further served to position individuals themselves as problematic, rather than the social structures and institutional processes that perpetuate risk and vulnerability. The concern for authors was that the resulting discourses reinforce narrow problematisations of health inequalities; have the potential to further stigmatise particular areas and population groups; and actually, as Blackman et al. (2012) describe, lead to a lack of critical reflection about how targeted interventions will reduce inequalities, beyond an assumption of benefit because they are ‘mainly supporting poorer people’.

Secondly, authors pointed to a plethora of governance arrangements stemming from both influential new public management and evidence-based policy and practice discourses, which further served to medicalise and individualise the problem of social inequalities in health within local health systems. For example, in England in 2001, explicit targets were introduced around reducing gaps in life expectancy and infant mortality. While initially not perceived to be a ‘high stakes issue’ in terms of accountability, increasing pressure to demonstrate rapid improvements against these targets was said to ultimately ‘bias’ action towards short-term ‘quick wins’ in the form of targeted pharmacological treatment for ‘at risk’ population groups (Blackman et al. 2012). Javanparast et al. (2018) have more recently described how long-term planning for action on the social determinants of health was constrained by a complex of funding arrangements, regulatory frameworks, reporting requirements, and timeframes that all aligned with taken-for-granted biomedical definitions of health and the dominant ‘curative’ paradigm within the Australian primary health care system. A further difficulty that individual actors faced in seeking to ‘redress the balance’ towards prevention and tackling health inequalities, was the inherently poor fit between interventions targeting the social determinants of health, and organisational imperatives to be able to predict, evidence, and quantify the direct impacts of investment (Orton et al. 2011). Indeed, even in more conducive contexts, where there was a greater recognition of the need to improve living conditions (e.g. Scotland), the challenge of making the ‘financial case’ for action, within these organisational frameworks, persisted (Blackman et al. 2012). Also highlighted were a number of practical constraints which were said to further limit action on the wider determinants of health. These included having the right knowledge and skill sets within local health systems to deliver longer-term cross-sectoral programmes of work (Javanparast et al. 2018), and, most notably in an English context, frequent health system reorganisations which continuously disrupt the working-relationships needed for long-term partnerships and action (Warwick‐Giles et al. 2017).

Lastly, studies pointed to an additional layer of alignment between internal organisational discourses and the external pressures which local health systems are under. Blackman et al. (2012), for example, outline how a focus on treatment reflects the ‘preoccupation’ with hospitals and acute care services amongst the media, elected representatives, and indeed the public. The distinct lack of ‘bottom-up’ pressure to prioritise health inequalities, coupled with the need for health system actors to be ‘apolitical’ when publicly advocating for health system or policy change (Raphael and Brassolotto 2015; Pauly et al. 2017) led to a persistent sense that health inequalities would always be easily ‘eclipsed’ or ‘overshadowed’ by more ‘politically sensitive’ priorities (Blackman et al. 2009).

### Challenges in operationalising counter-discourses

While authors did not explicitly label health equity discourses, or ideas around the social determinants of health, as ‘counter-discourses’ per se, they are undoubtedly designed to go ‘against the grain’ (Mead et al. 2020) and challenge dominant ways of thinking and working. There were however extensive difficulties reported by health system actors in actually trying to operationalise these ideas in practice, and not solely due to the challenges set out above. McIntyre et al. (2013), for example, describe how ideas around tackling the root causes of health inequalities (i.e. inequalities in power, money, and resources) were perceived by Canadian community and public health workers to be ‘overwhelming’, and, in light of the longer-term timeframes for impact, ‘offering little’ to people dealing day-to-day with populations experiencing inequality. These concerns were not limited to more front-line practitioners but were also expressed by health system leaders who similarly described how health equity and the social determinants of health often appeared in the dialogue ‘too big to tackle’ (Pauly et al. 2017), further reflecting that it seemed as though ‘health equity’ had become an ‘umbrella term’ which had ‘momentum’, but without the necessary ‘clarity’ to allow for its practical application in their everyday work. Indeed, even talking about health equity proved a challenge for some participants who felt that, across the health system, there wasn’t a shared understanding of the problem, nor a shared language to discuss it (Orton et al. 2011, Pauly et al. 2017). As a result, actors tended to revise their language to ensure that the way in which they framed health equity objectives were more readily accepted within their organisations and more easily understood. Examples included talking about vulnerable populations rather than complex social relations (Pauly et al. 2017), and talking about a ‘healthy place to live’ rather than the social and structural determinants of health (Raphael and Brassolotto 2015). Authors recognised that this more general process of ‘simplifying’ the problem of social inequalities in health was a ‘rational response’ and, an arguably inevitable consequence, for health system actors who have little power to influence the wider determinants (Mead et al. 2020). However, there were concerns that the ultimate consequence would be to further legitimise institutions norms (Javanparast et al. 2018), and reinforce the seemingly intractable nature of both health inequalities and wider social injustice (Pauly et al. 2017).

Importantly, despite all of the insights set out above about how powerful discourses constrain thinking and action, there were examples across the studies of individuals and organisations who were able to advocate for a structural perspective on social inequalities in health and enable health system engagement and action on their underlying causes. The fact that these individuals were often working within similar policy contexts, or with similar institutional constraints, led authors to conclude that the distinguishing factors were the ideas and beliefs held by senior staff and leaders who were able to ‘push against’ institutional discourses, norms, and mandates to promote new ways of thinking and working (Brassolotto et al. 2014; Javanparast et al. 2018). Warwick‐Giles et al. (2017), in particular, also highlighted the relational element and how shared histories of success and positive experiences of ‘doing things together’ in multi-organisational partnerships were essential in enabling collective action on health inequalities.

## Discussion

The purpose of this meta-ethnography was to synthesise qualitative research studies that have explored how ambitions to tackle health inequalities and their wider determinants take shape within local health systems. The included articles had a particular focus on illuminating how and why individual actors think about, and work to address, inequalities in health in the ways that they do. Drawing on a Foucauldian perspective, the resulting line-of-argument illustrates how health system actors are, as Bacchi (2009) would say, governed by ‘problematisations’. In particular, it has shown how the problem of social inequalities in health is moulded by powerful and influential discourses to fit both with pre-existing conceptions of health and inequalities, and the institutional practices which delimit what can be thought and done. In this section, I will first discuss how the findings of the meta-ethnography chime with insights generated through qualitative research in national policy settings, before discussing some recent critiques of health equity discourses and where they may be inadvertently contributing to the challenges outlined in the review.

Qualitative research with policy-makers has similarly emphasised the importance of the policy ‘problem’, and how it is invariably transformed to fit with both dominant discourses, and institutional structures and practices. Akin to the experience within local health systems, Qureshi (2013), for example, has illustrated how the drive for technical, quantifiable, and evidence-based solutions to health inequalities within the English civil service led to a ‘shifted conception’ of problem. While initially considered in terms of a social gradient that mandated action on the wider determinants of health, the problem, over the course of implementation, was quickly reimagined as ‘property’ of ‘deprived people or communities’, and one which could be addressed through an expansion of targeted health improvement interventions. Shifting conceptualisations of health inequalities have also been attributed to the more deliberate actions of civil servants who, in contrast to local health system actors, are especially constrained by the immediate political context, and the ideological persuasions of ministers. Drawing on insights from discursive institutionalism, Smith (2014) in particular, has highlighted how the task of ‘competitively marketing’ evidence and ideas within government led to actions on health inequalities being reframed in ways which were more politically palatable, in line with current policy directions, and, consequently, had the best chance of surviving the policy process. The end result, however, was that more challenging ideas, for example around reducing economic inequality, were significantly downplayed.

More recently, Lynch (2017) has provided a slightly different angle in explaining persistent policy inaction on the underlying causes of health inequalities. Based on qualitative interviews with policy-makers from across four European countries, she explains that the more fundamental issue is the way in which governments have embraced a reframing of social inequality *in health terms*. This reframing has had profound consequences for what the problem is understood be, where responsibility for action lies, and ultimately how amenable it is to change. As Lynch (2017) describes, a health framing inevitability leads to responsibility being situated within health ministries, where the problem of inequality becomes ‘medicalised’ by policy actors whose worldviews are more oriented towards individualism and a medical model of health. Importantly, while the ‘victim blaming’ discourses that Galvin (2002) has previously described were not to fore of studies included in this meta-ethnography, the legacy of emphasising individual responsibility for health and illness clearly does persist amongst health actors, and makes it difficult to move beyond a focus on individuals, to more explicitly consider the social structures and processes sustaining inequality. Smith (2013) has described how these difficulties are further compounded by institutional structures within departments, such as policy ‘silos’, which effectively serve to ‘filter’ out evidence and ideas that require cross-departmental working. The result is a focus within health departments on more immediately available levers, such as increasing health promotion activity and equitable access to services (Baum et al. 2013; Qureshi 2013; Smith 2013). Importantly, this challenge to the appropriateness of centring health, and health outcomes, is not limited to national policy settings. Ethnographic research within municipal government departments in Denmark found that a focus on *health* equity within intersectoral policy making led to the implementation of small-scale health promotion interventions in non-health settings (e.g. schools), rather than more co-ordinated action on the social determinants of health (Holt et al. 2017).

Highlighted in this meta-ethnography, and arguably further contributing to the institutional challenges already described, is the extent to which health equity ‘counter-discourses’ actually enable health system actors to think about inequalities in a more structural way. Indeed, many of the insights from the studies which focused specifically on actors’ perspectives on the social determinants of health, actually reflect critiques that have already been levelled at the model in wider literatures. For example, some authors would suggest that the predominant narrow, and reductionist, perspective is not surprising because the rainbow model itself promotes a focus on single discrete categories of determinants, rather than aspects of the political economy and wider social processes that shape their distribution (Hankivsky and Christoffersen 2008; Krieger et al. 2012, Yates‐Doerr 2020). To bring about this required shift in attention, Spiegel et al. (2015) have argued from a move away from talking about the social *determinants* of health, to better understand and theorise the social *determination* of health. In their recent reflection on 30 years of the rainbow model, Dahlgren and Whitehead (2021) pick up on these critiques and reiterate that, despite its popularity in the health equity field, the model is just a visual representation of the determinants of health, and not the determinants of health *inequalities.* They describe how latter involves a ‘further conceptual leap’ to consider different levels of power and resources; different levels of exposures to health hazards; differential impacts of the same exposures; life-course effects; and the social and economic impacts of being sick. As such, they conclude that there is a need to better illustrate these interconnected processes to enable effective action on the root causes of social inequalities in health. In light of the reflections from study participants in this review however, it is important that such resources enable constructive dialogue about tackling these root causes across health and wider local systems, and in a way that empowers and enables people to take action, rather than serving to overwhelming them.

Importantly, questions about the utility and impact of health equity ‘counter-discourses’ are not limited to professional groups, but are also increasingly being explored amongst different publics (Smith et al. 2021; Fairbrother et al. 2021; Lundell et al. 2013), with a view to understanding what is needed to generate the grassroots pressure to reduce inequalities which health system actors so often suggest is lacking. A recurrent finding in this research is the paradox between deep and nuanced understandings of the relationship between social inequality and poorer health outcomes, in particular amongst groups most exposed to social injustice, and a reluctance to acknowledge or accept the existence of a social gradient in health (Smith and Anderson 2017). To explain this paradox, authors have critiqued the determinism inherent in health equity discourses which, in relying on models of often unidirectional arrows from macro social structures to individual health outcomes, risk being both stigmatising and disempowering for different population groups (Lundell et al. 2013; Smith and Anderson 2017). This point was central to a recent critique of the social determinants of health where it was argued that the desire to avoid victim blaming within health equity discourses has actually served to downplay the role of individual agency to the point where people are effectively reduced to ‘puppets on strings’ (Lundberg 2020). These insights, and indeed more recent research with citizen juries in a UK context, point to a challenging balancing act in advancing health equity discourses which can simultaneously counter the problematic tendency for publics to individualise health, but in ways that are neither disempowering nor likely to reinforce prominent fatalistic discourses about the possibility for successfully reducing health inequalities (Smith et al. 2021).

## Conclusion

As Noblit and Hare (1988) describe, any meta-ethnographic account is but one possible interpretation of the phenomenon being studied and, indeed, the point is not to achieve ‘closure’, but rather to further enrich and enable discussion on a topic. The line-of-argument presented in this review centres upon the importance of understanding how problems take shape within systems, and illustrates how the problem of social inequalities in health is persistently transformed and reconfigured to fit both with pre-existing narrow and reductionist conceptions of health and inequality, and the institutional practices which constrain thinking and action within local health systems. This finding is especially important in light of current reforms in which local health systems, and their workforces, are increasing being drawn into conversations and planning to tackle social inequalities in health. It will be especially important to capture and understand how these cross-sectoral partnerships negotiate the influence of health systems, and the extent to which system leaders can ensure that narrower and more medicalised notions of health and inequity do not undermine the potential for more transformative action on the underlying causes of social inequalities in health.

## Supplementary Information

Below is the link to the electronic supplementary material.

Supplementary file1 (DOCX 55 kb)

## Data Availability

The data that supports the findings of this study are available from the author on request.
