# Supplementary material for: What shapes local health system actors’ thinking and action on social inequalities in health? A meta-ethnography
Source: Soc Theory Health. 2022 Jan 31;21(2):119–39. doi: 10.1057/s41285-022-00176-6 (PMC8801929; doi:10.1057/s41285-022-00176-6)
Supplement: Supplementary file 1 — Supplementary file1 (DOCX 55 kb) [file 41285_2022_176_MOESM1_ESM.docx]

**Online supplement**

**Appendix A: The eMERGe meta-ethnography reporting guidance**

**France, E. F., Cunningham, M., Ring, N., Uny, I., Duncan, E. A., Jepson, R. G., . . . Booth, A. (2019). Improving reporting of meta-ethnography: the eMERGe reporting guidance. BMC Medical Research Methodology, 19(1), 25.**

| No. | Criteria headings | Reporting criteria | Section in manuscript where reported |
| --- | --- | --- | --- |
| Phase 1: Selecting meta-ethnography and getting started | | | |
| Introduction | | | |
| 1 | Rationale and context for the meta-ethnography | Describe the gap in research or knowledge to be filled by the meta-ethnography, and the wider context of the meta-ethnography | Introduction |
| 2 | Aim(s) of the meta-ethnography | Describe the meta-ethnography aim(s) | Introduction |
| **3** | Focus of the meta-ethnography | Describe the meta-ethnography review question(s) (or objectives) | Introduction |
| **4** | Rationale for using meta-ethnography | Explain why meta-ethnography was considered the most appropriate qualitative synthesis methodology | Not described due to word count limit |
| Phase 2: Deciding what is relevant | | | |
| Methods | | | |
| **5** | Search strategy | Describe the rationale for the literature search strategy | Deciding what is relevant |
| **6** | Search processes | Describe how the literature searching was carried out and by whom | Deciding what is relevant |
| **7** | Selecting primary studies | Describe the process of study screening and selection, and who was involved | Deciding what is relevant |
| Findings | | | |
| **8** | Outcome of study selection | Describe the results of study searches and screening | Figure 1  Findings  Appendix B |
| Phase 3: Reading included studies | | | |
| Methods | | | |
| **9** | Reading and data extraction approach | Describe the reading and data extraction method and processes | Reading and data extraction |
| **10** | Presenting characteristics of included studies | Describe characteristics of the included studies | Table 1 |
| Phase 4: Determining how studies are related | | | |
| Methods | | | |
| **11** | Process for determining how studies are related | Describe the methods and processes for determining how the included  studies are related:  - Which aspects of studies were  compared  AND  - How the studies were compared | Analysis and synthesis |
| Findings | | | |
| **12** | Outcome of relating studies | Describe how studies relate to each other | Analysis and synthesis |
| Phase 5: Translating studies into one another | | | |
| Methods | | | |
| **13** | Process of translating studies | Describe the methods of translation:  - Describe steps taken to preserve the context and meaning of the relationships between concepts within and across studies  - Describe how the reciprocal and refutational translations  were conducted  - Describe how potential alternative interpretations or explanations were considered in the translations | Analysis and synthesis |
| Findings | | | |
| **14** | Outcome of translation | Describe the interpretive findings of the translation. | Table 2 |
| Phase 6: Synthesizing translations | | | |
| Methods | | | |
| **15** | Synthesis process | Describe the methods used to develop overarching concepts (“synthesised translations”)  Describe how potential alternative interpretations or  explanations were considered in the synthesis | Analysis and synthesis |
| Findings | | | |
| **16** | Outcome of synthesis process | Describe the new theory, conceptual framework, model, configuration, or interpretation of data developed from the synthesis | Findings |
| Phase 7: Expressing the synthesis | | | |
| Discussion | | | |
| **17** | Summary of findings | Summarize the main interpretive findings of the translation and synthesis and compare them to existing literature | Findings/Discussion and conclusion |
| **18** | Strengths, limitations, and reflexivity | Reflect on and describe the strengths and limitations of the synthesis:  - Methodological aspects—for example, describe how the synthesis findings were influenced by the nature of the included studies and how the meta-ethnography was conducted.  - Reflexivity—for example, the impact of the research team on the synthesis findings | Discussion |
| **19** | Recommendations and conclusions | Describe the implications of the synthesis | Discussion and conclusion |

**Appendix B: Excluded citations with reasons**

| **Reason** | **Excluded citations (n=1140)** |
| --- | --- |
| Not a peer-reviewed article | 11 |
| Not a qualitative study | 9 |
| Qualitative but not collecting in-depth data (e.g. survey) | 16 |
| Wrong sample (e.g. undergraduates) | 13 |
| Evaluating specific programme/service change | 27 |
| Evaluating training or capacity building | 8 |
| Focused on inequities in access to healthcare | 3 |
| Not an explanatory account of factors shaping thinking and action | 45 |
| Not in English | 5 |
| No access | 2 |
| Same content reported in included study in more depth | 1 |

**Not a peer-reviewed article (n = 11)**

1. Bodea Crisan AR. An exploration of the ideology of health promotion and critical implications for public health: ProQuest Information & Learning; 2014.
2. Byhoff E, Taylor LA. You can't scale unicorns: Exploring community based organizations' perspectives on health care's social determinants of health programming. Journal of General Internal Medicine. 2019;34 (2 Supplement):S416.
3. Clopton TM. Georgia government leaders' lived experience creating and implementing health equity policies. Minnesota: Walden University; 2020.
4. Cunningham BA, Johnson PJ, Rockwood T. Tackling health equity: leadership's perceptions of health care personnel and organizations. Journal of General Internal Medicine. 2014;29:S217-S8.
5. Cunningham BA, Scarlato A. Ensnared by color blindness: Discourse on healthcare disparities. Journal of General Internal Medicine. 2018;33 (2 Supplement 1):183.
6. James C, Johnson SR. 'We're trying to build the business case for achieving health equity'. Modern Healthcare. 2016;46(17):30-1.
7. Knight EK. Lost in translation: The professional public health discourse of poor health: ProQuest Information & Learning; 2012.
8. Lavin T, Metcalfe O. P62 Improving health equity via the social determinants of health in the EU. Journal of Epidemiology & Community Health. 2010;64:A57-8.
9. Paradiso de Sayu RR. Investigating and addressing social determinants of health in a community-based participatory research process: ProQuest Information & Learning; 2014.
10. Schaff KA. Local health departments engaging in policy change to achieve health equity: An examination of the foreclosure crisis: ProQuest Information & Learning; 2017.
11. Singhal R, Sreenivasan A, Zareef S, Kajita G. Perceptions of social determinants of health among international medical graduates: A qualitative study. Journal of General Internal Medicine. 2019;34(2 Supplement):S312.

**Not a qualitative study (n=9)**

1. Blackman T, Hunter D, Marks L, Harrington B, Elliott E, Williams G, et al. Wicked comparisons: Reflections on cross-national research about health inequalities in the UK. Evaluation. 2010;16(1):43-57.
2. Collins PA, Hayes MV. Twenty years since Ottawa and Epp: researchers' reflections on challenges, gains and future prospects for reducing health inequities in Canada. Health Promotion International. 2007;22(4):337-45.
3. Dean HD, Fenton KA. Integrating a Social Determinants of Health Approach into Public Health Practice: A Five-Year Perspective of Actions Implemented by CDC's National Center for HIV/AIDS, Viral Hepatitis, STD, and TB Prevention. Public Health Reports. 2013;128:5-11.
4. Johnson S, Abonyi S, Jeffery B, Hackett P, Hampton M, McIntosh T, et al. Recommendations for action on the social determinants of health: a Canadian perspective. Lancet. 2008;372(9650):1690-3.
5. Mockenhaupt R, Woodrum A. Developing Evidence for Structural Approaches to Build a Culture of Health: A Perspective From the Robert Wood Johnson Foundation. Health Education & Behavior. 2015;42(1):15S-9S.
6. Raphael D, Curry-Stevens A, Bryant T. Barriers to addressing the social determinants of health: Insights from the Canadian experience. Health Policy. 2008;88(2-3):222-35.
7. Sanders D, Sanders D. A global perspective on health promotion and the social determinants of health. Health Promotion Journal of Australia. 2006;17(3):165-7.
8. Shah UA, Hadayia JM, Forys LE. From Principles to Practice: One Local Health Department's Journey Toward Health Equity. Health Equity. 2017;1(1):23-7.
9. Wilkinson RG. The need for an interdisciplinary perspective on the social determinants of health. Health Economics. 2000;9(7):581-3.

**Qualitative but not collecting in-depth data (e.g. survey) (n=16)**

1. Blackman T, Dunstan K. Qualitative Comparative Analysis and Health Inequalities: Investigating Reasons for Differential Progress with Narrowing Local Gaps in Mortality. Journal of Social Policy. 2010;39:359-73.
2. Blackman T, Wistow J, Byrne D. A Qualitative Comparative Analysis of factors associated with trends in narrowing health inequalities in England. Social Science & Medicine. 2011;72(12):1965-74.
3. Collins PA. Do great local minds think alike? Comparing perceptions of the social determinants of health between non-profit and governmental actors in two Canadian cities. Health Education Research. 2012;27(3):371-84.
4. Collins PA, Abelson J, Eyles JD. Knowledge into action? Understanding ideological barriers to addressing health inequalities at the local level. Health Policy. 2007;80(1):158-71.
5. Collins PA, Hayes MV. Examining the Capacities of Municipal Governments to Reduce Health Inequities: A Survey of Municipal Actors' Perceptions in Metro Vancouver. Canadian Journal of Public Health-Revue Canadienne De Sante Publique. 2013;104(4):E304-E10.
6. França VH, Confalonieri UEC. Local communities, health and the sustainable development goals: the case of Ribeirão das Neves, Brazil. Cadernos Metrópole. 2016;18(36):365-75.
7. Hatch SL, Frissa S, Verdecchia M, Stewart R, Fear NT, Reichenberg A, et al. Identifying socio-demographic and socioeconomic determinants of health inequalities in a diverse London community: the South East London Community Health (SELCoH) study. BMC Public Health. 2011;11(1):861-.
8. Knight EK. Shifting Public Health Practice to Advance Health Equity: Recommendations From Experts and Community Leaders. Journal of Public Health Management and Practice. 2014;20(2):188-96.
9. Lawless A, Lane A, Lewis FA, Baum F, Harris P. Social determinants of health and local government: understanding and uptake of ideas in two Australian states. Australian and New Zealand Journal of Public Health. 2017;41(2):204-9.
10. Lynam MJ. Does discourse matter? Using critical inquiry to engage in knowledge development for practice. Primary Health Care Research and Development. 2007;8(1):54-67.
11. Palacio A, Seo D, Medina H, Singh V, Suarez M, Tamariz L. Provider Perspectives on the Collection of Social Determinants of Health. Population Health Management. 2018;21(6):501-8.
12. Popay J, Kowarzik U, Mallinson S, Mackian S, Barker J. Social problems, primary care and pathways to help and support: addressing health inequalities at the individual level. part I: the GP perspective. Journal of Epidemiology and Community Health. 2007;61(11):966-71.
13. Raphael D. A discourse analysis of the social determinants of health. Critical Public Health. 2011;21(2):221-36.
14. Suarez-Balcazar Y, Mirza MP, Garcia-Ramirez M. Health disparities: Understanding and promoting healthy communities. Journal of Prevention & Intervention in the Community. 2018;46(1):1-6.
15. Wolf L, Vigna AJ, Inzeo PT, Ceraso M, Wolff T. From Roots to Results: A Qualitative Case Study of the Evolution of a Public Health Leadership Institute Building Capacity in Collaborating for Equity and Justice. Health Education & Behavior. 2019;46(1 Supplement):33S-43S.
16. Zaboli R, Tourani S, Hesam Seyedin S, Oliaie Manesh A. Prioritizing the determinants of social-health inequality in Iran: A multiple attribute decision making application. Iranian Red Crescent Medical Journal. 2014;16 (4) (no pagination)(e12607).

**Wrong sample (e.g. undergraduates) (n=13)**

1. Baum FE, Laris P, Fisher M, Newman L, MacDougall C. "Never mind the logic, give me the numbers": Former Australian health ministers' perspectives on the social determinants of health. Social Science & Medicine. 2013;87:138-46.
2. Baverstock KA, Gargya DM, Jackson M, Stupans I. Identifying Future Health Professionals' Understanding of the Determinants of Health. The Journal of Nursing Education. 2018;57(12):756-9.
3. Damari B, Oveisi S, Azizkhani N. A model for utilizing social determinants of health approach by faculty members. [Persian]. Koomesh. 2018;20(2):366-74.
4. Easterling D, McDuffee L. Becoming Strategic: Finding Leverage Over the Social and Economic Determinants of Health. Foundation Review. 2018;10(1):90-+.
5. Garthwaite K, Smith KE, Bambra C, Pearce J. Desperately seeking reductions in health inequalities: perspectives of UK researchers on past, present and future directions in health inequalities research. Sociology of Health & Illness. 2016;38(3):459-78.
6. Gostelow N, Barber J, Gishen F, Berlin A. Flipping social determinants on its head: Medical student perspectives on the flipped classroom and simulated patients to teach social determinants of health. Medical Teacher. 2018;40(7):728-35.
7. Lisitza A, Wolbring G. EcoHealth and the Determinants of Health: Perspectives of a Small Subset of Canadian Academics in the EcoHealth Community. International Journal of Environmental Research and Public Health. 2018;15(8).
8. Manhire-Heath R, Cormack D, Wyeth E. '...but I just prefer to treat everyone the same...': general practice receptionists talking about health inequities. Australian Journal of Primary Health. 2019;25(5):430-4.
9. McKay K. An exploration of student midwives' perceptions of health promotion in contemporary practice. MIDIRS Midwifery Digest. 2008;18(2):165-74.
10. Reno RPMSWMA, Beaujolais BMSWMA, Davis TSPM. Facilitating mechanisms for integrating care to promote health equity across the life course: reflections from social work trainees. Social Work in Health Care. 2018:1-15.
11. Russell RG, Davidson H, Rhoads C, Petrusa ER. How do we understand the determinants of health? An exploration of distributed knowledge and interprofessional health sciences education. Journal of Interprofessional Care. 2017;31(1):118-21.
12. Smith KE. The politics of ideas: The complex interplay of health inequalities research and policy. Science and Public Policy. 2014;41(5):561-74.
13. Stewart M, Reutter L, Makwarimba E, Veenstra G, Love R, Raphael D. Left out: Perspectives on social exclusion and inclusion across income groups. Health Sociology Review. 2008;17(1):78-94.

**Evaluating specific programme/service change (n=27)**

1. Baum F, Delany-Crowe T, MacDougall C, Lawless A, van Eyk H, Williams C. Ideas, actors and institutions: lessons from South Australian Health in All Policies on what encourages other sectors' involvement. BMC Public Health. 2017;17.
2. Blanchet Garneau A, Browne AJ, Varcoe C. Understanding competing discourses as a basis for promoting equity in primary health care. BMC Health Services Research. 2019;19:1-11.
3. Brown M, Karatzias T, O'Leary L. The health role of local area coordinators in Scotland: a mixed methods study. Journal of Intellectual Disabilities. 2013;17(4):387-402.
4. Carlisle S. Tackling health inequalities and social exclusion through partnership and community engagement? A reality check for policy and practice aspirations from a Social Inclusion Partnership in Scotland. Critical Public Health. 2010;20(1):117-27.
5. de Sayu RP, Sparks SM. Factors That Facilitate Addressing Social Determinants of Health Throughout Community-based Participatory Research Processes. Progress in Community Health Partnerships. 2017;11(2):119-27.
6. Erasmus E, Gilson L, Govender V, Nkosi M. Organisational culture and trust as influences over the implementation of equity-oriented policy in two South African case study hospitals. International Journal for Equity in Health. 2017;16.
7. Fosse E, Helgesen MK. How can local governments level the social gradient in health among families with children? The case of Norway. International Journal of Child Youth & Family Studies. 2015;6(2):328-46.
8. Fosse E, Sherriff N, Helgesen M. Leveling the Social Gradient in Health at the Local Level: Applying the Gradient Equity Lens to Norwegian Local Public Health Policy. International Journal of Health Services. 2019;49(3):538-54.
9. Green J, Edwards P. The limitations of targeting to address inequalities in health: A case study of road traffic injury prevention from the UK. Critical Public Health. 2008;18(2):175-87.
10. Hall RL, Jacobson PD. Examining Whether The Health-In-All-Policies Approach Promotes Health Equity. Health Affairs. 2018;37(3):364-70.
11. Hassen N, Tyler I, Manson H. Influence of revised public health standards on health equity action: a qualitative study in Ontario, Canada. International Journal for Equity in Health. 2017;16.
12. Jean-Jacques M, Mahmud Y, Hamil J, Kang R, Duckett P, Yonek JC. Lessons Learned About Advancing Healthcare Equity From the Aligning Forces for Quality Initiative. American Journal of Managed Care. 2016;22(12):S413-S22.
13. McPherson C, Ndumbe-Eyoh S, Betker C, Oickle D, Peroff-Johnston N. Swimming against the tide: A Canadian qualitative study examining the implementation of a province-wide public health initiative to address health equity. International Journal for Equity in Health. 2016;15.
14. Mundo W, Manetta P, Fort MP, Sauaia A. A Qualitative Study of Health in All Policies at the Local Level. Inquiry: The Journal of Health Care Organization, Provision, and Financing. 2019;56:1-7.
15. Porroche-Escudero A, Popay J. The Health Inequalities Assessment Toolkit: supporting integration of equity into applied health research. Journal of Public Health. 2020;23.
16. Powell K, Thurston M, Bloyce D. Theorising lifestyle drift in health promotion: explaining community and voluntary sector engagement practices in disadvantaged areas. Critical Public Health. 2017;27(5):554-65.
17. Rankin D, Backett-Milburn K, Platt S. Practitioner perspectives on tackling health inequalities: Findings from an evaluation of healthy living centres in Scotland. Social Science & Medicine. 2009;68(5):925-32.
18. Ravaghi H, Goshtaei M, Olyaee Manesh A, Abolhassani N, Arabloo J. Stakeholders' perspective on health equity and its indicators in Iran: a qualitative study. Medical journal of the Islamic Republic of Iran. 2015;29:250-.
19. Riggs E, Block K, Warr D, Gibbs L. Working better together: new approaches for understanding the value and challenges of organizational partnerships. Health Promotion International. 2014;29(4):780-93.
20. Scheele CE, Little I, Diderichsen F. Governing health equity in Scandinavian municipalities: The inter-sectorial challenge. Scandinavian Journal of Public Health. 2018;46(1):57-67.
21. Schmidt M, Joosen I, Kunst AE, Klazinga NS, Stronks K. Generating Political Priority to Tackle Health Disparities: A Case Study in the Dutch City of The Hague. American Journal of Public Health. 2010;100:S210-S5.
22. Steele LS, Lemieux-Charles L, Clark JP, Glazier RH. The impact of policy changes on the health of recent immigrants and refugees in the inner city - A qualitative study of service providers' perspectives. Canadian Journal of Public Health-Revue Canadienne De Sante Publique. 2002;93(2):118-22.
23. Steer M, Machin AI. A qualitative exploration of stakeholder perceptions of the implementation of place-based working and its potential to reduce health inequality. Journal of Public Health. 2018; 40(4):813-819.
24. Storm I, Aarts MJ, Harting J, Schuit AJ. Opportunities to reduce health inequalities by 'Health in All Policies' in the Netherlands: An explorative study on the national level. Health Policy. 2011;103(2-3):130-40.
25. Synnevag ES, Amdam R, Fosse E. Legitimising Inter-Sectoral Public Health Policies: A Challenge for Professional Identities? International Journal of Integrated Care. 2019;19(4).
26. Tebb KP, Pica G, Twietmeyer L, Diaz A, Brindis CD. Innovative Approaches to Address Social Determinants of Health Among Adolescents and Young Adults. Health equity. 2018;2(1):321-8.
27. Tenbensel T, Cumming J, Ashton T, Barnett P. Where there's a will, is there a way?: Is New Zealand's publicly funded health sector able to steer towards population health? Social Science & Medicine. 2008;67(7):1143-52.

**Evaluating training or capacity building (n=8)**

1. Cusack C, Cohen B, Mignone J, Chartier MJ, Lutfiyya Z. Reorienting Public Health Nurses' Practice With a Professional Practice Model. Canadian Journal of Nursing Research. 2017;49(1):16-27.
2. Folker AP, Lauridsen S. Using action learning to reduce health inequity in Danish municipalities. Leadership in Health Services (1751-1879). 2017;30(2):194-207.
3. Gugglberger L, Sherriff N, Davies J, Broucke S. Building capacity to reduce health inequalities through health promotion in Europe. Journal of Public Health. 2016;24(1):73-81.
4. Henschke N, Mirny A, Haafkens JA, Ramroth H, Padmawati S, Bangha M, et al. Strengthening capacity to research the social determinants of health in low-and middle-income countries: lessons from the INTREC programme. BMC Public Health. 2017;17.
5. Levesque M, Levine A, Bedos C. Humanizing Oral Health Care through Continuing Education on Social Determinants of Health: Evaluative Case Study of a Canadian Private Dental Clinic. Journal of Health Care for the Poor and Underserved. 2016;27(3):971-92.
6. MacVicar R, Williamson A, Cunningham DE, Watt G. What are the CPD needs of GPs working in areas of high deprivation? Report of a focus group meeting of 'GPs at the Deep End'. Education for Primary Care. 2015;26(3):139-45.
7. Naz A, Rosenberg E, Andersson N, Labonte R, Andermann A, Collaboration C. Health workers who ask about social determinants of health are more likely to report helping patients Mixed-methods study. Canadian Family Physician. 2016;62(11):E684-E93.
8. Signal L, Martin J, Reid P, Carroll C, Howden-Chapman P, Ormsby VK, et al. Tackling health inequalities: moving theory to action. International Journal for Equity in Health. 2007;6:12-.

**Focused on inequities in access to healthcare (n=3)**

1. Cunningham BA, Scarlato ASM. Ensnared By Colorblindness: Discourse On Health Care Disparities. Ethnicity & Disease. 2018;28:235-40.
2. Lane H, Sturgess T, Philip K, Markham D, Walsh J, Hubbard W, et al. How Do Allied Health Professionals Define and Apply Equity When Making Resource Allocation Decisions? International Journal of Health Services. 2018;48(2):349-64.
3. Nkunu V, McLaughlin KJ. Why do some physicians choose to tackle inequities in healthcare? International Journal for Equity in Health. 2018;17(1):81.

**Not an explanatory account of factors shaping thinking and action (n=45)**

1. Blane DN, Hesselgreaves H, McLean G, Lough M, Watt GC. Attitudes towards health inequalities amongst GP trainers in Glasgow, and their ideas for changes in training. Education for primary care: an official publication of the Association of Course Organisers, National Association of GP Tutors, World Organisation of Family Doctors. 2013;24(2):97-104.
2. Browne GR, Davern M, Giles-Corti B. 'Punching above their weight': a qualitative examination of local governments' organisational efficacy to improve the social determinants of health. Australian and New Zealand Journal of Public Health. 2019;43(1):81-87.
3. Carr SM, Clarke CL. The manager's role in mobilizing and nurturing development: entrenched and engaged approaches to change. Journal of Nursing Management. 2010;18(3):332-8.
4. Cohen D, Huynh T, Sebold A, Harvey J, Neudorf C, Brown A. The population health approach: A qualitative study of conceptual and operational definitions for leaders in Canadian healthcare. SAGE Open Medicine. 2014;2:2050312114522618.
5. Dalton S, Orford J, Parry J, Laburn-Peart K. Three ways of talking about health in communities targeted for regeneration - Interviews with community professionals. Journal of Health Psychology. 2008;13(1):65-78.
6. de Andrade M. Tackling health inequalities through asset-based approaches, co-production and empowerment: ticking consultation boxes or meaningful engagement with diverse, disadvantaged communities? Journal of Poverty and Social Justice. 2016;24(2):127-41.
7. De Oliveira A, Chavannes B, Steinecker M, Denantes M, Chastang J, Ibanez G. How French general practitioners adapt their care to patients with social difficulties? Family Medicine and Community Health. 2019;7(4).
8. Exworthy M, Berney L, Powell M. 'How great expectations in Westminster may be dashed locally': the local implementation of national policy on health inequalities. Policy & Politics. 2002;30(1):79-96.
9. Figueroa C, Kadkhoda F, Miranda J, Kataoka SH, Bromley E, Wells KB, et al. Community Partnering for Behavioral Health Equity: Public Agency and Community Leaders' Views of its Promise and Challenge. Ethnicity & Disease. 2018;28:397-406.
10. Firoz T, Vidler M, Tatenda Makanga P, Boene H, Chiaú R, Sevene E, et al. Community perspectives on the determinants of maternal health in rural southern Mozambique: a qualitative study. Reproductive Health. 2016;13:123-31.
11. Freeman T, Baum F, Lawless A, Gwyn J, Labonte R, Bentley M, et al. Reaching those with the greatest need: how Australian primary health care service managers, practitioners and funders understand and respond to health inequity. Australian Journal of Primary Health. 2011;17(4):355-61.
12. Freeman T, Baum F, Lawless A, Javanparast S, Jolley G, Labonte R, et al. Revisiting the ability of Australian primary healthcare services to respond to health inequity. Australian Journal of Primary Health. 2016;22(4):332-8.
13. Girgis L, Van Gurp G, Zakus D, Andermann A. Physician experiences and barriers to addressing the social determinants of health in the Eastern Mediterranean Region: a qualitative research study. BMC Health Services Research. 2018;18.
14. Grier SA, Schaller TK. Operating in a Constricted Space: Policy Actor Perceptions of Targeting to Address US Health Disparities. Journal of Public Policy & Marketing. 2020;39(1):31-47.
15. Grimm MJTN, Helgesen MK, Fosse E. Reducing social inequities in health in Norway: Concerted action at state and local levels? Health Policy. 2013;113(3):228-35.
16. Kapiriri L, Martin DK. Successful Priority Setting in Low and Middle Income Countries: A Framework for Evaluation. Health Care Analysis. 2010;18(2):129-47.
17. Kataoka SH, Figueroa C, Bromley E, Patel H, Wells KB, Ijadi-Maghsoodi R, et al. Stakeholder Perspectives on the Social Determinants of Mental Health in Community Coalitions. Ethnicity & Disease. 2018;28:389-96.
18. Kneale D, Rojas-Garcia A, Thomas J. Obstacles and opportunities to using research evidence in local public health decision-making in England. Health Research Policy and Systems. 2019;17(1):61.
19. Lammertsen RC, Tong S, Nguyen AT, Lee V. Health equity message framing: a Canadian regional health authority perspective. International Journal of Health Promotion and Education. 2017;55(2):81-95.
20. Mabhala MA. Health inequalities as a foundation for embodying knowledge within public health teaching: a qualitative study. International Journal for Equity in Health. 2013;12.
21. Mabhala MA. Public health nurse educators' conceptualisation of public health as a strategy to reduce health inequalities: A qualitative study. International Journal for Equity in Health. 2014;14.
22. Marks L, Hunter DJ, Scalabrini S, Gray J, McCafferty S, Payne N, et al. The return of public health to local government in England: changing the parameters of the public health prioritization debate? Public Health. 2015;129(9):1194-203.
23. Marks L, Weatherly H, Mason A. Prioritizing investment in public health and health equity: what can commissioners do? Public Health. 2013;127(5):410-8.
24. Mason C, Orr J, Harrisson S, Moore R. Health professionals' perspectives on service delivery in two Northern Ireland communities. Journal of Advanced Nursing. 1999;30(4):827-34.
25. McCauley MP, Ramanadhan S, Viswanath K. Assessing opinions in community leadership networks to address health inequalities: a case study from Project IMPACT. Health Education Research. 2015;30(6):866-81.
26. McCollum R, Theobald S, Otiso L, Martineau T, Karuga R, Barasa E, et al. Priority setting for health in the context of devolution in Kenya: implications for health equity and community-based primary care. Health Policy and Planning. 2018;33(6):729-42.
27. Morrison J, Pons-Vigues M, Becares L, Burstrom B, Gandarillas A, Dominguez-Berjon F, et al. Health inequalities in European cities: Perceptions and beliefs among local policymakers. BMJ Open. 2014;4 (5) (no pagination)(e004454).
28. Morrison J, Pons-Vigues M, Diez E, Pasarin MI, Salas-Nicas S, Borrell C. Perceptions and beliefs of public policymakers in a Southern European city. International Journal for Equity in Health. 2015;14.
29. Mumtaz Z, Salway S, Bhatti A, Shanner L, Zaman S, Laing L, et al. Improving maternal health in Pakistan: toward a deeper understanding of the social determinants of poor women's access to maternal health services. American Journal of Public Health. 2014;104 Suppl 1:S17-24.
30. Narain KDC, Zimmerman FJ, Richards J, Fielding JE, Cole BL, Teutsch SM, et al. Making Strides Toward Health Equity: The Experiences of Public Health Departments. Journal of Public Health Management and Practice. 2019;25(4):342-7.
31. Pedersen PV, Hjelmar U, Hoybye MT, Rod MH. Can inequality be tamed through boundary work? A qualitative study of health promotion aimed at reducing health inequalities. Social Science & Medicine. 2017;185:1-8.
32. Petticrew M, Platt S, McCollam A, Wilson S, Thomas S, Petticrew M, et al. "We're not short of people telling us what the problems are. We're short of people telling us what to do": an appraisal of public policy and mental health. BMC Public Health. 2008;8(1):314-.
33. Petticrew M, Whitehead M, Macintyre SJ, Graham H, Egan M. Evidence for public health policy on inequalities: 1: The reality according to policymakers. Journal of Epidemiology and Community Health. 2004;58(10):811-6.
34. Raphael D, Brassolotto J, Baldeo N. Ideological and organizational components of differing public health strategies for addressing the social determinants of health. Health Promotion International. 2015;30(4):855-67.
35. Rooddehghan Z, ParsaYekta Z, Nasrabadi AN. Equity in nursing care: A grounded theory study. Nursing ethics. 2019;26(2):598-610.
36. Roussy V, Livingstone C. Surviving neoliberalism, maintaining values: Community health mergers in Victoria, Australia. International Journal of Health Planning & Management. 2018;33(2):e636-e47.
37. Ryan-Ibarra S, Nishimura H, Gallington K, Grinnell S, Bekemeier B. Time to Modernize: Local Public Health Transitions to Population-Level Interventions. Journal of Public Health Management and Practice. 2019;11.
38. Sheridan NF, Kenealy TW, Connolly MJ, Mahony F, Barber PA, Boyd MA, et al. Health equity in the New Zealand health care system: a national survey. International Journal for Equity in Health. 2011;10.
39. Sheridan NF, Kenealy TW, Schmidt-Busb JIG, Rea HH. Population health in New Zealand 2000-2013: From determinants of health to targets. SAGE Open Medicine. 2015;3:2050312115573654.
40. Smith MJ, Thompson A, Upshur REG. Is 'health equity' bad for our health? A qualitative empirical ethics study of public health policy-makers' perspectives. Canadian Journal of Public Health = Revue Canadienne de Sante Publique. 2018;109(5-6):633-42.
41. Sunderland N, Harris P, Johnstone K, Del Fabbro L, Kendall E. Exploring health promotion practitioners' experiences of moral distress in Canada and Australia. Global Health Promotion. 2015;22(1):32-45.
42. Turner D, Salway S, Mir G, Ellison GT, Skinner J, Carter L, et al. Prospects for progress on health inequalities in England in the post-primary care trust era: professional views on challenges, risks and opportunities. BMC Public Health. 2013;13:274.
43. Warr DJ, Mann R, Kelaher M. 'A lot of the things we do ... people wouldn't recognise as health promotion': addressing health inequalities in settings of neighbourhood disadvantage. Critical Public Health. 2013;23(1):95-109.
44. Whitehead M, Petticrew M, Graham H, Macintyre SJ, Bambra C, Egan M. Evidence for public health policy on inequalities: 2: Assembling the evidence jigsaw. Journal of Epidemiology and Community Health. 2004;58(10):817-21.
45. Yelland J, Riggs E, Dawson W, Vanpraag D, Szwarc J, Brown S. 'It requires something drastic': Interviews with health care leaders about organisational responses to social disadvantage. Women and Birth. 2020;S1871-5192(19)30919-9.

**Not in English (n = 5)**

1. Cotichelli G, Di Meo G, Giampieri P. Knowledge and common sense of nurses on the health's inequities: an exploratory study. Professioni infermieristiche. 2016;69(3):167-73.
2. Figueiredo DS, Heidemann ITSB, Fernandes GCM, Arawaka AM, de Oliveira LS, Magagnin AB. Health promotion to social determinants: possibility for equity. Journal of Nursing UFPE / Revista de Enfermagem UFPE. 2019;13(4):943-51.
3. Gonzalez Rojo E, Alvarez-Dardet C, Lopez Fernandez LA, Grp Trabajo Salud P. Towards a social determinants-oriented approach to public health: workshop report. Gaceta Sanitaria. 2018;32(5):488-91.
4. Plaza Tesias A, Sole Brichs C. Consensus process in the field of health inequalities. The Delphi technique. [Spanish]. Atencion primaria / Sociedad Espanola de Medicina de Familia y Comunitaria. 1995;16(4):207-10.
5. Sanz E, Esnaola S, Aldasoro E, Bacigalupe A, Zuazagoitia J. Steps towards the institutionalization of health impact assessment in Spain: perception of public health professionals. Anales Del Sistema Sanitario De Navarra. 2012;35(3):403-12.

**No access (n=2)**

1. Kim M-H, 박여리, 김새롬. Knowledge Translation for Health Inequality in South Korea. Health and Social Science. 2017;44:45-80.
2. Spiteri K, McScat HA, Calleja N, Mamo J. Spatial health inequalities-health professionals perspective 2011 Malta. European Journal of Public Health. 2012;22:242-3.

**Same content reported in included study in more depth (n=1)**

1. Harrington BE, Smith KE, Hunter DJ, Marks L, Blackman TJ, McKee L, et al. Health inequalities in England, Scotland and Wales: stakeholders' accounts and policy compared. Public health. 2009;123(1):e24-8.
